# The genetic relationships between different definitions of emissions outputs and feedlot production traits

**DOI:** 10.1186/s12711-026-01086-1

**Published:** 2026-09-11

**Authors:** M. D. Madsen, T. Granleese, R. Hergenhan, H. Aliloo, M. N. Aldridge, S. A. Clark

**Affiliations:** 1 School of Environmental and Rural Science, University of New England, Armidale, NSW 2351 Australia; 2 Genetics, Agriculture and Biosecurity, Department of Primary Industries and Regional Development, Grafton, NSW 2460 Australia; 3 Animal Genetics and Breeding Unit, University of New England, Armidale, NSW 2351 Australia

## Abstract

**Background:**

Selective breeding is a potential strategy for reducing methane (CH_4_) emissions in ruminants. In Australian beef cattle, large scale emission recording is currently being conducted using GreenFeed Emission Monitors (C-Lock Inc, USA). GreenFeeds repeatedly collect CH_4_ and carbon dioxide (CO_2_) spot-samples of varying duration on attending animals. This study used emission phenotypes from Australian feedlot beef cattle to compare variance components and predictive ability of 14 different datasets. The datasets differed in the minimum duration (two- or three minutes) required of spot-samples, and the minimum number of spot-samples (one, five, ten, 15, 20, 25 or 30) required of animals, to be included in the analysis. For each data requirement, variance components were estimated for trial average CH_4_ and CO_2_ production and CH_4_ efficiency traits, including residual CH_4_ adjusted for either feed intake, average daily gain or mid-test weight; CH_4_ intensity (CH_4_/mid-test weight); and CH_4_ yield (CH_4_/daily feed intake). This study also examined the genetic relationships between these emission traits and feed intake, liveweight and growth in Australian feedlot beef cattle.

**Results:**

Including animals with at least five spot-samples of two minutes or longer duration, when calculating trial average emission traits, performed as well as datasets with a higher minimum number of spot-samples, and better than datasets including animals with a lower minimum number of spot-samples. The required minimum duration of included spot-samples did not have systematic impacts on either variance components or predictive ability. Under the data requirement of a minimum of five, two-minute or longer spot-samples, the heritabilities of the emission traits were 0.24–0.43 (SE 0.06–0.08), with higher heritability of the emission production traits than the CH_4_ efficiency traits. Feed intake, live weight and growth were moderately positive genetically correlated to CH_4_ production (0.39–0.60, SE 0.08–0.14), moderately negative to moderately positive genetically correlated to the CH_4_ efficiency traits (-0.27 to 0.26, SE 0.08–0.19), and moderately to highly positive genetically correlated to CO_2_ production (0.54–0.88, SE 0.05–0.12).

**Conclusions:**

This study showed that requiring animals to have a minimum of five spot-samples of at least two-minutes duration are appropriate data requirements for the emission data currently available on Australian feedlot beef cattle. The results further showed that feed intake, growth and liveweight had the strongest genetic relationships with CO_2_ production, followed by CH_4_ production, and lastly the CH_4_ efficiency traits.

**Supplementary Information:**

The online version contains supplementary material available at https://doi.org/10.1186/s12711-026-01086-1.

## Background

Enteric methane (CH_4_) is a substantial source of greenhouse gas emissions from ruminant livestock species [1]. Consequently, multiple abatement strategies for CH_4_ emissions from ruminants are being explored; selective breeding of animals with genetically lower CH_4_ emissions being one of those strategies [2, 3]. The cumulative and permanent nature of selective breeding means it is possible to reduce CH_4_ emissions from livestock across consecutive generations of selection.

In beef cattle, CH_4_ is commonly measured as CH_4_ production (g/day), which has been found to have significant genetic variance and low- to moderate heritabilities (0.09–0.43) [4–8]. This shows that selection to reduce CH_4_ emission is feasible. However, methane production has been found to have highly positive genetic correlations to live weight (0.28–0.83) and feed intake (0.57–0.84) [4, 6–8]. These genetic correlations need to be considered when implementing selection against CH_4_ in beef cattle, particularly to avoid reducing live weight.

In order to account for the relationship between the CH_4_ production of animals and the animals’ intake or live weight, CH_4_ efficiency traits are often derived from CH_4_ production. Previously studied CH_4_ efficiency traits include CH_4_ intensity, which is the ratio of CH_4_ production to an output such as liveweight, and CH_4_ yield, which is the ratio of CH_4_ production to an input such as feed intake [4–6]. Methane intensity and yield are thus expressions of how much CH_4_ is emitted per unit of in- or output. Another type of CH_4_ efficiency trait is residual CH_4_, which is obtained by adjusting the CH_4_ production for in- and/or outputs, such as feed intake or liveweight [4–6]. This means residual CH_4_ traits are measures of the amount of CH_4_ that is not due to the weight, growth and/or feed intake of the animal.

Due to CH_4_ efficiency traits accounting, at least partially, for the relationship between CH_4_ and feed intake or live weight, they tend to have lower genetic correlations to feed intake and live weight than CH_4_ production. The genetic correlations between these CH_4_ efficiency traits and feed intake or live weight have been reported as moderately negative to moderately positive (-0.44-0.45) [4, 6, 7]. Methane efficiency traits also tend to have lower heritabilities than the CH_4_ production trait they are derived from. Studies examining both CH_4_ production and efficiency reported heritabilities of 0.15–0.25 for the CH_4_ efficiency traits compared to 0.30–0.31 for CH_4_ production [4, 6, 7].

While a lot of research on emissions from ruminants have focused on CH_4_, some studies have also examined carbon dioxide (CO_2_) production and its relationship to CH_4_ production, feed intake and live weight. Moderately to highly positive genetic correlations have been reported between CO_2_ production and CH_4_ production (0.60–0.75) [7, 8] and live weight (0.28–0.96) [7]. Meanwhile CO_2_ production has been shown to be highly positively genetically correlated to feed intake (0.78–0.95) [7–9]. The strong relationship between CO_2_ production and feed intake has received particular attention due to the possibility of using emissions recording as a way of obtaning feed intake proxy traits [7–9]. This is especially relevant for pasture grazing systems where directly recording individual feed intake is even more challenging than recording emissions production.

GreenFeed Emission Monitor units (C-Lock Inc, USA) are often used to record emissions in beef cattle. GreenFeed is an automated feeder system that monitors CH_4_ and CO_2_ fluxes from breath and eructation gas from cattle. The GreenFeed works by spot-sampling animals when they voluntarily access the machine. The voluntary nature of GreenFeed recording means that animals measured using such a system have a variable amount of information available [10]. Previous studies have suggested that limiting data to spot-samples of greater than three minutes in duration and requiring animals to have at least 30 spot-samples is necessary to obtain repeatable phenotypic records [11].

For genetic evaluation, limiting animals to a specific number of spot-samples would likely result in removing a substantial amount of information that could otherwise have been utilised. While breeding values can be obtained from animals without phenotypes, through their genetic or genomic relationship with phenotyped animals [12–13], the breeding values of the animals are expected to be more accurate if their own phenotypes are included in the evaluation. Similarly, spot-samples of less than three-minute duration are an information source that could potentially be utilised for breeding value estimation. On the other hand, including spot-samples of shorter duration and/or animals with fewer spot-samples could also add unnecessary noise to the phenotypes, adversely impacting estimation of variance components and the prediction ability of the models. It is therefore worthwhile to re-evaluate the data requirements particularly in the context of genetic evaluation.

The aims of this study were; (1) to examine the appropriate minimum number and duration of spot-samples for calculating individual trial average emission traits for estimation of variance components and breeding values of different emission traits using GreenFeed records, and (2) to provide estimates of variance components of emission traits and the genetic relationship between emission traits, body weight, growth and feed intake in Australian feedlot beef cattle steers.

## Methods

### Data

The feed intake, weight and emissions traits used in this study were recorded between October 2022 and May 2024 at Tullimba Feedlot (1831 Torryburn Road, Torryburn, NSW 2358, Australia). The recorded animals originated from two projects conducted in Australia: (1) the Southern Multibreed project (SMB) and (2) the Angus Sire Benchmarking Program (ASBP). The projects were approved by the New South Wales Department of Primary Industries & Regional Development (SMB: ARA-OAEC-0309) and/or the University of New England Animal Ethics Committees (SMB: ARA21-072, ARA24-051; ASBP: AEC20-071, ARA23-060). All animals in the project were managed according to the Australian Code for the Care and Use of Animals for Scientific Purposes (NHMRC, 2013).

The SMB dataset used in this study consisted of 1403 steers from five different breeds (Angus, Hereford, Charolais, Shorthorn and Wagyu) that were raised together in multi-breed contemporary groups [14]. The SMB steers were measured in 24 groups of 31–90 head (average group size of 65, median group size of 71). All 24 groups contained Angus steers as well as animals from at least one other *Bos taurus* breed. Meanwhile, the ASBP dataset used in this study consisted of 542 purebred Angus steers trialled in 9 single-breed groups of 22–91 head (average group size of 64, median group size of 68). Animals from the two reference populations were recorded simultaneously, and were managed by the same staff under the same protocols.

### Phenotyping

Upon arriving at Tullimba feedlot the animals were given an adjustment period of two to six weeks prior to starting their 70-day feed efficiency testing period. The adjustment period allowed the animals to acclimatise to the feeders and to the total mixed ration (TMR). The same ration and feeding schedule were used for all animals.

The TMR, fed during the feed intake and emission recording period, consisted of tempered barley (66.3%), cottonseed (10%), hay (10%), millrun pellets (2.5%), a liquid supplement (5.2%) and water (6%) and was mixed onsite from purchased ingredients. The ingredients were delivered in large batches to reduce variability, and tempering and mixing were adjusted, as needed, to reach target moisture and nutrient composition and to reduce variation across batches. The TMR target composition was 75% dry matter and 12% protein with a metabolizable energy content of 12 MJ ME/kg dry matter. The liquid supplement was formulated by Performance Feeds and delivered in bulk and contained molasses, monensin, vitamins, minerals and micronutrients to ensure animals’ mineral and vitamin requirements were met. Feed analyses were conducted regularly to ensure targets were being met (within limits). Mixer testing occurred to ensure the ration was mixed homogenously for delivery to cattle.

The animals started their feed efficiency trial at an average age of 517 days (SD of 58 days, range of 377–805 days). Daily feed intake (DFI) was collected using the Vytelle Sense™ individual animal data capture system [15], which measured individual feed intake as the weight difference of the individual feed bunk before and after an animal visits the bunk. Animal visitations were registered by radio frequency ID tags. Live weights were recorded manually on all steers six times with ~ 14-day intervals starting at day 0 of the feed intake recording period. Slight variation between exact day of each weighing existed between groups for practical reasons such as labour availability, access to facilities and prevailing weather conditions. The live weights were also used to calculate average daily gain (ADG) over the feed efficiency test.

Methane and CO_2_ production were recorded using GreenFeed units. The GreenFeed unit measured the concentration of CH_4_ and CO_2_ (ppm) in an animal’s breath when the animal visited the machine. The emissions production (g/day) was then calculated by C-Lock Inc from the emissions concentrations. C-Lock’s processing protocol does not allow for processing of emissions from visits of shorter than two-minute duration. To entice the animals to stay two minutes or longer in the machine, visiting animals were rewarded with multiple drops of pelleted feed (on average 35 g per drop, range 28–47 g per drop). Pellets were a purchased commercial product (Pryde’s EasiRide^®^) with target specifications of 10% DM, 11.6 MJME/kg and a minimum of 12% protein and as such were similar to the TMR but novel enough to be attractive to the cattle. To ensure that the machines were not monopolised by a few of the animals, animals were allowed a maximum of eight cup drops per visit and had a minimum interval of five hours between visits within a single day.

One to four GreenFeed units were used per group resulting in a stocking density of around 20–40 head per machine. For 6–15 days prior to the emission data collection the cattle were trained to access the GreenFeed units. Each machine had a race attached to the front of the machine consisting of a pair of 2.1 m long cattle panels. During the training period, the races at the machines had a gap of 1.3 m to desensitise cattle to voluntary claustrophobia. Once the majority of animals in a group were consistently attending the GreenFeed units the races were narrowed to 0.7 m to eliminate multiple steers accessing the machines at once. The official recording period began on the day following the races being narrowed. The emissions trait recording occurred at varying stages during the feed intake recording period but was never started before the feed-acclimation period had finished. The majority of groups were tested for emissions after two weeks of feed efficiency testing. Some variation existed in days on trial due to GreenFeed availability, resulting in recording ranging from 17 to 82 days on trial with an average of 41 days on trial.

### Quality control

Quality control was carried out for all phenotypes and included removing all records from animals with unknown breed, date of birth or genotype, removing records that deviated more than three SDs from the individual mean or mean of the animals of the same breed in the same contemporary group (CG; concatenation of birth year, birth location, rearing group, and recording group), ensuring CG sizes of a minimum five animals, and ensuring CGs contained offspring of more than one sire. Lastly, one trial group had all CO_2_ spot-samples removed due to the group average CO_2_ exceeding the dataset average CO_2_ by more than three SDs. No trial groups exhibited average CH_4_ outside the overall mean CH_4_ ± three SDs.

After quality control, 1808 animals from 329 sires were retained (Table 1 for breed distribution). Of the 1808 animals with at least one phenotype recorded, 1469 animals had at least one CH_4_ and one CO_2_ spot-sample as well as a DFI, ADG and weight phenotype.

**Table 1:** Number of animals with phenotypes by breed

Trait		Angus	Hereford		Charolais	Shorthorn	Wagyu	Total
Daily feed intake	Animals	963	303	141		200	173	1780
	Daily Records	66,839	21,007	9873		14,122	11,991	123,832
Weight	Animals*	975	307	141		203	175	1801
Methane	Animals	905	266	124		144	106	1460
	Spot-samples	58,295	16,525	6865		5338	4938	91,835
Carbon dioxide	Animals	877	252	124		144	98	1460
	Spot-samples	56,580	15,416	6890		5365	4328	91,677
**Total #animals**		980	308	142		203	175	1808
**Total #sires**		143	65	38		37	39	322

*With all six weight records for the feed intake trial

### Correction of emission spot-samples

**Fig. 1:**
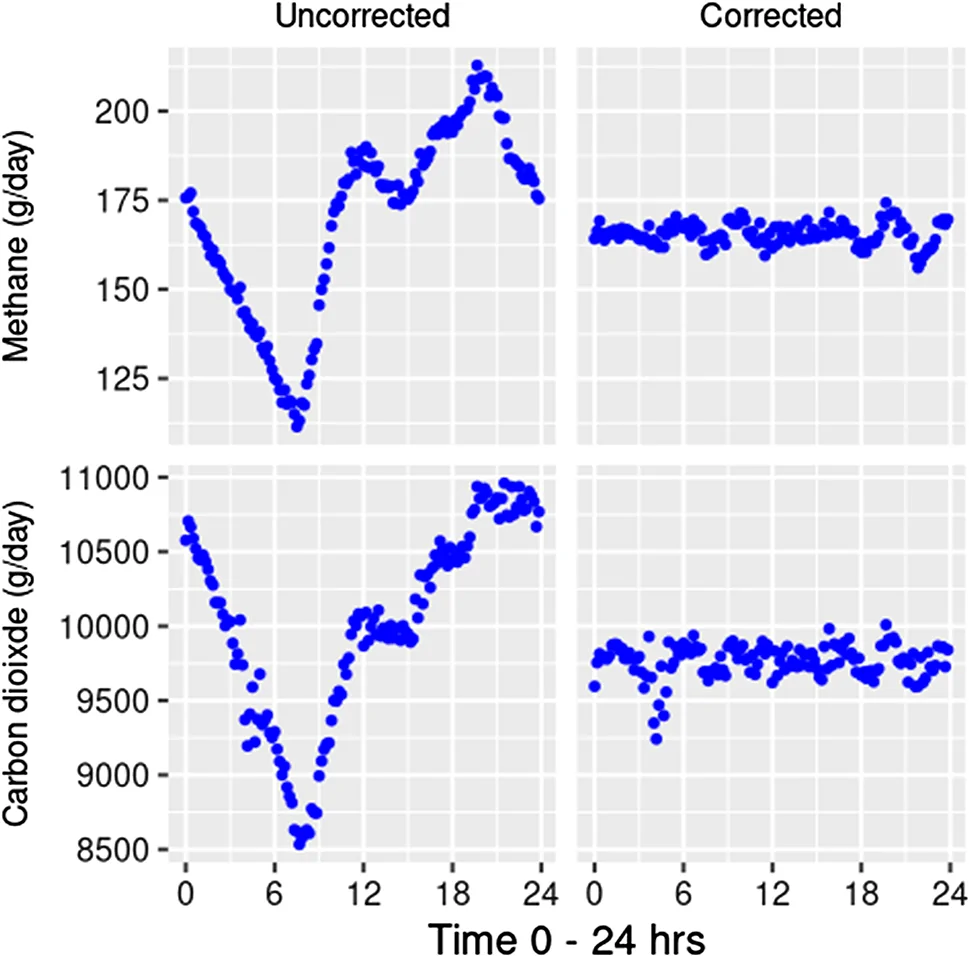
Phenotypic averages within 10-minute intervals (blue dots) across time of day for methane (top) and carbon dioxide (bottom) production before (left) and after (right) correcting for the diurnal pattern

After quality control, the emissions spot-samples needed to be corrected for the time of recording due to the diurnal pattern of emissions (Fig. 1). The diurnal pattern is linked to feeding behaviour, generally showing a sharp increase in emissions directly after feeding followed by a slow decrease until the next meal [16–20]. Biased average phenotypes can occur if spot-samples of emissions are not corrected for the diurnal pattern. For example, an animal with a habit of visiting the GreenFeed before the morning feeding would have low average emission production, while an animal that consistently visits the GreenFeed one to two hours after consuming feed would have high average emission production. The adjustment of emissions records for the time of day (essentially accounting for the diurnal pattern) allows for more equal comparison of animals recorded over the day.

A generalised additive model was used to correct for the diurnal pattern. While correcting for the diurnal pattern, the spot-samples were also corrected for the fixed effect of GreenFeed unit to account for systematic differences between spot-samples recorded with different machines. Thus, the model followed:

1 $$\:y=\:\mu\:+\:GreenFeedUnit+s\left(Time\right)+e$$

Where * y* was the uncorrected phenotype, $\:\mu\:$ was the phenotypic mean, * GreenFeedUnit* was the unit by which the spot-sample was recorded, * Time* was the time of day in seconds fitted with a smoothing function ($\:s\left(x\right)$), and * e* was the residuals. The corrected phenotypes were then:

2 $$\:{y}_{c}=\mu\:+e$$

The corrected phenotypes showed a clear correction for the diurnal pattern (Fig. 1).

### Traits for analysis

Seven emission traits and three production traits were defined to be analysed in this study. Daily feed intake was calculated as the average daily feed intake in kg (total mixed ration and GreenFeed pellets) within animal over the 70-day feed efficiency trial. Average daily gain (ADG) was calculated as the regression coefficient of an individual animals’ linear regression of weight in kg over days in the feed efficiency trial. Mid-test weight (MWT) was defined as weight on day 0 of the feed efficiency trial plus ADG multiplied by the days between the start of the feed efficiency testing period and the mid-day of the emission testing period.

To examine the trial average emission production, CH_4_ and CO_2_ production (CH_4−Prd_ and CO_2−Prd_) were defined as the trial average of the corrected emission phenotypes (spot-samples corrected for the diurnal pattern and GreenFeed unit). Due to the large SD of CO_2_ (2106 g/day), corrected CO_2_ was converted from g/day to kg/day (SD 2.1 kg/day) prior to calculating CO_2−Prd_.

Trial average CH_4_ intensity (CH_4−Int_), CH_4_ yield (CH_4−Yld_) and three different residual CH_4_ traits (CH_4−ResDFI_, CH_4−ResADG_ and CH_4−ResMWT_) were defined to investigate CH_4_ efficiency. CH_4−Int_ was calculated as the ratio of CH_4−Prd_ to MWT, while CH_4−Yld_ was calculated as the ratio of CH_4−Prd_ to DFI. The residual CH_4_ traits were obtained using (co)variances and genetic regression coefficients obtained from bivariate animal models with CH_4−Prd_ and either DFI, ADG or MWT (see “Statistical analysis – Bivariate models for residual methane traits” below for more details) such that the residual traits were genetically independent from the production trait [21]. This approach means that the CH_4−Res_ traits do not have a phenotype. Furthermore, using genetic regressions means the CH_4−Res_ traits represented the trial average CH_4_ production that was genetically independent of either DFI, ADG or MWT. This independence means that the genetic correlation between a residual trait obtained using genetic regressions and the trait(s) regressed on is zero and consequently selection on the residual trait would not cause a correlated response in the trait(s) used for the regression. Table 2 provides descriptive statistics of the analysed traits.

**Table 2:** Descriptive statistics

Trait	Unit	Mean (SD)	Range
DFI	kg/day	15.43 (2.25)	5.67–22.45
ADG	kg/day	1.70 (0.39)	0.24–4.60
MWT	kg	567.28 (79.84)	267.08-844.02
CH_4-Prd_	g/day	208.18 (35.77)	84.60–336.98
CH_4-Int_	(g/day)/kg MWT	0.37 (0.06)	0.15–0.64
CH_4-Yld_	(g/day)/(kg DFI)	13.32 (2.34)	6.56–36.28
CO_2-Prd_	kg/day	10.45 (1.52)	4.84–17.57

DFI = daily feed intake, ADG = average daily gain, MWT = mid-test weight, CH_4-Prd_ = methane production, CH_4-Int_ = methane intensity, CH_4-Yld_ = methane yield, CO_2-Prd_ = carbon dioxide production

### Genotypes

All animals were genotyped as part of their respective projects. As part of the SMB project, 6247 animals were genotyped using Neogen GGP Bovine 100 K BeadChip (2024 animals) and Zoetis ZBU medium density 50 K SNP arrays (4223 animals). For both arrays, the genotype calls with a score below 0.15 were treated as missing [22]. Further quality control was applied separately to each array using PLINK 1.9 [23], excluding SNPs and animals with a call rate lower than 90%, monomorphic SNPs across all animals, and SNPs located on the sex chromosomes (X and Y). After quality control, Fimpute 3 [24] was used to impute sporadically missing genotypes across the two SNP arrays and to create a common SNP set for all animals. Animals from the same population were used as the reference for imputing missing genotypes. In total, 109,429 SNPs and 8055 animals were retained.

Additionally, a second dataset including 1109 Angus cattle from the ASBP with 61,105 SNPs was obtained through the Angus Australia Genotype QC and Imputation Pipeline [25]. Only the SNP common between the SMB and ASBP datasets were kept, resulting in 9164 animals, including 3872 Angus, 967 Charolais, 1883 Hereford, 1103 Shorthorn and 1249 Wagyu cattle, and 52,095 SNPs used in this study. The mapping information for all markers was based on ARS-UCD 1.2 [26] bovine genome assembly.

### Investigating the impact of different data requirements

To determine the impact of different data requirements on the estimation of variance components and breeding values for the emission traits, multiple datasets were constructed. The datasets differed in minimum duration of sampling values (two- and three minutes) and minimum number of spot-samples per animal (one, five, ten, 15, 20, 25 or 30) resulting in a total of 14 datasets. The minimum of two-minute duration is the default lower limit set by C-Lock Inc. The requirement of a minimum of 30 spot-samples of three-minute or longer duration was suggested for obtaining repeatable phenotypes by Arthur et al. [11] and was considered the highest standard in this study. The emission traits defined above were calculated within each of the 14 datasets (Additional file 1, Table S1 for descriptive statistics of the emission traits in the 14 datasets).

### Cross-validation subsets

For each of the 14 datasets, five cross-validation subsets were created. The cross-validation involved masking the phenotypes of 200 animals. The 200 masked animals had at least one two-minute spot-sample of both CH_4_ and CO_2_ and a MWT, ADG and DFI phenotype and could not be masked more than once but were otherwise chosen at random.

As the masked animals were only required to have at least one two-minute or longer duration spot-sample the number of masked animals was 200 in the datasets where animals were required to have at least one two-minute or longer duration spot-sample but varied between the remaining 13 datasets. Across all datasets, 12–14% of the animals were masked in the cross-validation.

### Statistical analysis

#### Univariate models

Variance components, their associated standard errors and genomic estimated breeding values (GEBVs) for CH_4−Prd_, CH_4−Int_, CH_4−Yld_, and CO_2−Prd_ for each of the 14 datasets were obtained using univariate models fitted in BlupF90+ [27]. Univariate models were also used to obtain variance components and GEBVs for DFI, ADG and MWT.

The fixed effects of breed (5 classes), CG and age at emission trial start (days) were included for all traits. Age of dam at birth (years) was included in the analysis of MWT. Age of dam was not available for 25 of the animals with MWT, and these animals were therefore removed from the analysis. Age of dam was not found significant for the other traits, when fitting a linear mixed model with sires as the random effect using the lme4 package [28] in R [29].

The general univariate model followed:

$$\:\mathbf{y}=\mathbf{X}\mathbf{b}+\mathbf{Z}\mathbf{a}+\mathbf{e}$$

where **y** was a vector of the phenotypes (CH_4−Prd_, CH_4−Int_, CH_4−Yld_, CO_2−Prd_, DFI, ADG or MWT), **b** was a vector of fixed effects, **a** and **e** were random additive genetic animal effects (i.e. GEBVs) and residuals, respectively, and **X** and **Z** were the design matrices linking animals to their fixed effect classes and their genotypes, respectively.

The additive genetic animal effect was assumed normally distributed with $\mathbf{a} \sim N\left(\mathbf{0}, \sigma_a^2 \otimes \mathbf{G}\right)$ where $\:{{\upsigma\:}}_{\mathrm{a}}^{2}$ was the additive genetic and residual variance, and **G** was the genomic relationship matrix. The genomic relationship matrix was constructed following:

3 $$\:\mathbf{G}=\frac{\mathbf{M}\mathbf{M}^{\prime\:}}{{\sum\:}_{k=1}^{m}2{p}_{k}{q}_{k}}$$

where $\:\mathbf{M}$ was the matrix for additive marker covariates and contained $\:0-2{p}_{k}$, $\:1-2{p}_{k}$, and $\:2-2{p}_{k}$ for AA, AB, and BB genotypes, respectively; $\:{p}_{k}$ was the frequency of allele B at marker *k* and $\:{q}_{k}=1-{p}_{k}$ [30].

The residuals were normally distributed with $\mathbf{e} \sim N\left(\mathbf{0}, \mathbf{W}\sigma_{\in}^2\right)$, where $\:{\sigma\:}_{\in\:}^{2}$ was a scaling variance, and $\:\mathbf{W}$ was a diagonal matrix. For the emission traits, the diagonal elements of $\:\mathbf{W}$ was the weight placed on the individual phenotypes. Multiple functions based on the number of spot-samples ($\:{n}_{s}$) used to calculate the trial average were tested (Additional file 2 Figure S1) and $\:{w}_{ii}=1-\frac{1}{{n}_{s,i}+1}$ was used for this study. The residual variance was then calculated as $\:{\sigma\:}_{e}^{2}=\frac{{{\upsigma\:}}_{\in\:}^{2}}{\overline{diag\left(\mathbf{W}\right)}}$. For DFI, ADG and MWT, $\:\mathbf{W}=\mathbf{I}$, meaning $\:\overline{diag\left(\mathbf{W}\right)}=1$ and thus $\:{\sigma\:}_{e}^{2}={\sigma\:}_{\in\:}^{2}$.

The univariate models were also fitted to the five cross-validation subsets within each dataset in order to obtain GEBVs of the masked animals. The predictive performance was compared, within trait across different datasets, based on the bias ($\:{{\upmu\:}}_{\mathrm{w},\mathrm{p}}$) and dispersion ($\:{\mathrm{b}}_{\mathrm{w},\mathrm{p}}$) of the GEBVs of the masked animals obtained using the whole dataset (subscript ‘w’) and partial data (subscript ‘p’) following the LR method [31]. The bias was standardised to genetic SD estimated in the whole dataset ($\:{{\upsigma\:}}_{\mathrm{a},\mathrm{w}}$).

4 $$\:{\mu\:}_{w,p}=\frac{{\overline{\mathbf{a}}}_{\mathbf{p}}-{\overline{\mathbf{a}}}_{\mathbf{w}}}{{{\upsigma\:}}_{\mathrm{a},\mathrm{w}}}$$

5 $$\:{b}_{\mathrm{w},\mathrm{p}}=\frac{\mathrm{c}\mathrm{o}\mathrm{v}\left({\mathbf{a}}_{\mathbf{w}},{\mathbf{a}}_{\boldsymbol{p}}\right)}{\mathrm{v}\mathrm{a}\mathrm{r}\left({\mathbf{a}}_{\mathbf{p}}\right)}$$

In addition, the accuracy of predictions ($\:{\rho\:}_{\mathrm{p},\mathrm{w}}$) (sometimes termed stability [31]) was defined as:

6 $$\:{\rho\:}_{\mathrm{p},\mathrm{w}}=\frac{\mathrm{c}\mathrm{o}\mathrm{v}\left({\mathbf{a}}_{\mathbf{p}},{\mathbf{a}}_{\mathbf{w}}\right)}{\sqrt{\mathrm{v}\mathrm{a}\mathrm{r}\left({\mathbf{a}}_{\mathbf{p}}\right)\mathrm{v}\mathrm{a}\mathrm{r}\left({\mathbf{a}}_{\mathbf{w}}\right)}}$$

These parameters were calculated for each of the five cross-validation subsets and the average and SD across the subsets were calculated. The difference in bias, dispersion and accuracy of GEBVs were used to assess the data requirements impact on the estimation of breeding values.

### Bivariate models for residual methane traits

To obtain (co)variance components and GEBVs for CH_4−ResDFI_, CH_4−ResADG_ and CH_4−ResMWT_ bivariate models were fitted following:

$$\begin{aligned}\:\left[\begin{array}{c}{\mathbf{y}}_{\mathbf{E}\mathbf{P}}\\\:{\mathbf{y}}_{\mathbf{P}\mathbf{T}}\end{array}\right]&=\left[\begin{array}{cc}{\mathbf{X}}_{\mathbf{E}\mathbf{P}}&\:0\\\:0&\:{\mathbf{X}}_{\mathbf{P}\mathbf{T}}\end{array}\right]\left[\begin{array}{c}{\mathbf{b}}_{\mathbf{E}\mathbf{P}}\\\:{\mathbf{b}}_{\mathbf{P}\mathbf{T}}\end{array}\right]\cr&\quad+\left[\begin{array}{cc}{\mathbf{Z}}_{\mathbf{E}\mathbf{P}}&\:0\\\:0&\:{\mathbf{Z}}_{\mathbf{P}\mathbf{T}}\end{array}\right]\left[\begin{array}{c}{\mathbf{a}}_{\mathbf{E}\mathbf{P}}\\\:{\mathbf{a}}_{\mathbf{P}\mathbf{T}}\end{array}\right]\cr&\quad+\left[\begin{array}{c}{\mathbf{e}}_{\mathbf{E}\mathbf{P}}\\\:{\mathbf{e}}_{\mathbf{P}\mathbf{T}}\end{array}\right]\end{aligned}$$

where the subscript EP refers to CH_4−Prd_ and the subscript PT refers to the production trait (DFI, ADG or MWT). The distribution assumptions for the random effects were

$$\begin{bmatrix}a_{\mathrm{EP}} \\a_{\mathrm{PT}}\end{bmatrix}\sim N\left(\begin{bmatrix}0 \\0\end{bmatrix},\begin{bmatrix}\sigma_{a_{\mathrm{EP}}}^2 &\sigma_{a_{\mathrm{EP}},a_{\mathrm{PT}}} \\\sigma_{a_{\mathrm{EP}},a_{\mathrm{PT}}} &\sigma_{a_{\mathrm{PT}}}^2\end{bmatrix}\otimes G\right)$$  

for the additive genetic animals effects and

$$\begin{bmatrix}e_{\mathrm{EP}} \\e_{\mathrm{PT}}\end{bmatrix}\sim N\left(\begin{bmatrix}0 \\0\end{bmatrix},\begin{bmatrix}W_{\mathrm{EP}} & 0 \\0 & W_{\mathrm{PT}}\end{bmatrix}\begin{bmatrix}\sigma_{\epsilon_{\mathrm{EP}}}^2 &\sigma_{\epsilon_{\mathrm{EP}},\epsilon_{\mathrm{PT}}} \\\sigma_{\epsilon_{\mathrm{EP}},\epsilon_{\mathrm{PT}}} &\sigma_{\epsilon_{\mathrm{PT}}}^2\end{bmatrix}\right)$$

for the residuals, where $\:{{\upsigma\:}}_{{\mathrm{a}}_{\mathrm{E}\mathrm{P}},{\mathrm{a}}_{\mathrm{P}\mathrm{T}}}$ is the additive genetic covariance between CH_4−Prd_ and the production trait and $\:{{\upsigma\:}}_{{\epsilon}_{\mathrm{E}\mathrm{P}},{\epsilon}_{\mathrm{P}\mathrm{T}}}$ is the residual covariance between CH_4−Prd_ and the production trait. All other effects were as described for the univariate models.

The residual emission traits (CH_4−Res_) were obtained as the distribution of GEBVs of CH_4−Prd_ conditioning on the GEBVs of DFI, ADG or MWT using genetic regression coefficients, such that the genetic correlation between the CH_4−Res_ trait and the production trait was zero [21].

The genetic regression coefficient was calculated as:

7 $$\:{\upbeta\:}=\frac{{{\upsigma\:}}_{{\mathrm{a}}_{\mathrm{E}\mathrm{P}},{\mathrm{a}}_{\mathrm{P}\mathrm{T}}}}{{{\upsigma\:}}_{{\mathrm{a}}_{\mathrm{P}\mathrm{T}}}^{2}}$$

and the genetic (co)variance matrix of the CH_4−Res_, CH_4−Prd_ and production trait was obtained from:

8 $$\begin{aligned}\:\left[\begin{array}{ccc}{{\upsigma\:}}_{{\mathrm{a}}_{\mathrm{R}\mathrm{e}\mathrm{s}}}^{2}&\:{{\upsigma\:}}_{{\mathrm{a}}_{\mathrm{R}\mathrm{e}\mathrm{s}},{\mathrm{a}}_{\mathrm{E}\mathrm{P}}}&\:{{\upsigma\:}}_{{\mathrm{a}}_{\mathrm{R}\mathrm{e}\mathrm{s}},{\mathrm{a}}_{\mathrm{P}\mathrm{T}}}\\\:{{\upsigma\:}}_{{\mathrm{a}}_{\mathrm{R}\mathrm{e}\mathrm{s}},{\mathrm{a}}_{\mathrm{E}\mathrm{P}}}&\:{{\upsigma\:}}_{{\mathrm{a}}_{\mathrm{E}\mathrm{P}}}^{2}&\:{{\upsigma\:}}_{{\mathrm{a}}_{\mathrm{E}\mathrm{P}},{\mathrm{a}}_{\mathrm{P}\mathrm{T}}}\\\:{{\upsigma\:}}_{{\mathrm{a}}_{\mathrm{R}\mathrm{e}\mathrm{s}},{\mathrm{a}}_{\mathrm{P}\mathrm{T}}}&\:{{\upsigma\:}}_{{\mathrm{a}}_{\mathrm{P}\mathrm{T}},{\mathrm{a}}_{\mathrm{E}\mathrm{P}}}&\:{{\upsigma\:}}_{{\mathrm{a}}_{\mathrm{P}\mathrm{T}}}^{2}\end{array}\right]\\=\left[\begin{array}{cc}1&\:-{\upbeta\:}\\\:1&\:0\\\:0&\:1\end{array}\right]\:\left[\begin{array}{cc}{{\upsigma\:}}_{{\mathrm{a}}_{\mathrm{E}\mathrm{P}}}^{2}&\:{{\upsigma\:}}_{{\mathrm{a}}_{\mathrm{E}\mathrm{P}},{\mathrm{a}}_{\mathrm{P}\mathrm{T}}}\\\:{{\upsigma\:}}_{{\mathrm{a}}_{\mathrm{P}\mathrm{T}},{\mathrm{a}}_{\mathrm{E}\mathrm{P}}}&\:{{\upsigma\:}}_{{\mathrm{a}}_{\mathrm{P}\mathrm{T}}}^{2}\end{array}\right]\left[\begin{array}{ccc}1&\:1&\:0\\\:-{\upbeta\:}&\:0&\:1\end{array}\right]\end{aligned}$$

where $\:{{\upsigma\:}}_{{\mathrm{a}}_{\mathrm{R}\mathrm{e}\mathrm{s}}}^{2}$ was the additive genetic variance of the CH_4−Res_ trait (CH_4−ResDFI_, CH_4−ResADG_ or CH_4−ResMWT_), $\:{{\upsigma\:}}_{{\mathrm{a}}_{\mathrm{R}\mathrm{e}\mathrm{s}},{\mathrm{a}}_{\mathrm{E}\mathrm{P}}}$ was the genetic covariance between the CH_4−Res_ trait and CH_4−Prd_, and $\:{{\upsigma\:}}_{{\mathrm{a}}_{\mathrm{R}\mathrm{e}\mathrm{s}},{\mathrm{a}}_{\mathrm{P}\mathrm{T}}}$ was the genetic covariance between the CH_4−Res_ trait and production trait.

Similarly, the phenotypic (co)variance matrix was obtained using:

9 $$\begin{aligned}\:\left[\begin{array}{ccc}{{\upsigma\:}}_{{\mathrm{P}}_{\mathrm{R}\mathrm{e}\mathrm{s}}}^{2}&\:{{\upsigma\:}}_{{\mathrm{P}}_{\mathrm{R}\mathrm{e}\mathrm{s}},{\mathrm{P}}_{\mathrm{E}\mathrm{P}}}&\:{{\upsigma\:}}_{{\mathrm{P}}_{\mathrm{R}\mathrm{e}\mathrm{s}},{\mathrm{P}}_{\mathrm{P}\mathrm{T}}}\\\:{{\upsigma\:}}_{{\mathrm{P}}_{\mathrm{R}\mathrm{e}\mathrm{s}},{P}_{\mathrm{E}\mathrm{T}}}&\:{{\upsigma\:}}_{{\mathrm{P}}_{\mathrm{E}\mathrm{P}}}^{2}&\:{{\upsigma\:}}_{{\mathrm{P}}_{\mathrm{E}\mathrm{P}},{\mathrm{P}}_{\mathrm{P}\mathrm{T}}}\\\:{{\upsigma\:}}_{{\mathrm{P}}_{\mathrm{R}\mathrm{e}\mathrm{s}},{\mathrm{P}}_{\mathrm{P}\mathrm{T}}}&\:{{\upsigma\:}}_{{\mathrm{P}}_{\mathrm{P}\mathrm{T}},{\mathrm{e}}_{\mathrm{E}\mathrm{P}}}&\:{{\upsigma\:}}_{{\mathrm{P}}_{\mathrm{P}\mathrm{T}}}^{2}\end{array}\right]\cr=\left[\begin{array}{cc}1&\:-{\upbeta\:}\\\:1&\:0\\\:0&\:1\end{array}\right]\:\left[\begin{array}{cc}{{\upsigma\:}}_{{\mathrm{P}}_{\mathrm{E}\mathrm{P}}}^{2}&\:{{\upsigma\:}}_{{\mathrm{P}}_{\mathrm{E}\mathrm{P}},{\mathrm{P}}_{\mathrm{P}\mathrm{T}}}\\\:{{\upsigma\:}}_{{\mathrm{P}}_{\mathrm{E}\mathrm{P}},{\mathrm{P}}_{\mathrm{P}\mathrm{T}}}&\:{{\upsigma\:}}_{{\mathrm{P}}_{\mathrm{P}\mathrm{T}}}^{2}\end{array}\right]\left[\begin{array}{ccc}1&\:1&\:0\\\:-{\upbeta\:}&\:0&\:1\end{array}\right]\end{aligned}$$

where $\:{{\upsigma\:}}_{{\mathrm{P}}_{\mathrm{R}\mathrm{e}\mathrm{s}}}^{2}$, $\:{{\upsigma\:}}_{{\mathrm{P}}_{\mathrm{R}\mathrm{e}\mathrm{s}},{\mathrm{P}}_{\mathrm{E}\mathrm{P}}}$ and $\:{{\upsigma\:}}_{{\mathrm{P}}_{\mathrm{R}\mathrm{e}\mathrm{s}},{\mathrm{P}}_{\mathrm{P}\mathrm{T}}}$ were the phenotypic variance of the CH_4−Res_, the phenotypic covariance between the CH_4−Res_ trait and CH_4−Prd_, and the phenotypic covariance between the CH_4−Res_ trait and production trait, respectively.

As for the univariate models, variance components (and SEs) were estimated using BlupF90+ [27] and comparison of variance components and heritabilities across the 14 datasets was done.

The GEBVs of the residual emission traits ($\:{\mathbf{a}}_{\mathbf{R}\mathbf{E}\mathbf{P}}$) were calculated as:

10 $$\:{\mathbf{a}}_{\mathbf{R}\mathbf{E}\mathbf{P}}={\mathbf{a}}_{\mathbf{E}\mathbf{P}}-{\mathbf{a}}_{\mathbf{P}\mathbf{T}}\boldsymbol{*}{\upbeta\:}$$

The bivariate models were also fitted to the five cross-validation subsets within each dataset in order to obtain GEBVs for the CH_4−Res_ traits of the masked animals in each subset. The bias, dispersion and accuracy were then calculated based on the GEBVs of the masked animals within each cross-validation subset and averaged over the five subsets.

### Multi-trait models

Multi-trait models were used to obtain genetic correlations between all analysed traits. Generally bivariate models were used except when genetic correlations were estimated between CH_4−Int_, CH_4_-_Yld_, or CO_2−Prd_ and the CH_4−Res_ traits, and amongst the CH_4−Res_ traits themselves, where trivariate models were required.

The multi-trait models were only run on the dataset with the minimum requirements for duration of recording and number of spot-samples per animal determined to be sufficient by the comparison of variance components and predictive ability between the different data requirements scenarios (as described previously) for each trait. The general *m*-trait model followed:

$$\begin{bmatrix}y_1 \\\vdots \\y_m\end{bmatrix}=\begin{bmatrix}X_1 & & 0 \\& \ddots & \\0 & & X_m\end{bmatrix}\begin{bmatrix}b_1 \\\vdots \\b_m\end{bmatrix}+\begin{bmatrix}Z_1 & & 0 \\& \ddots & \\0 & & Z_m\end{bmatrix}\begin{bmatrix}a_1 \\\vdots \\a_m\end{bmatrix}+\begin{bmatrix}e_1 \\\vdots \\e_m\end{bmatrix}$$

The models fitted the same fixed effects for each trait as described for the univariate models, while the random additive genetic and residual effects were fitted with assumed distributions of: $$\begin{bmatrix}a_1 \\\vdots \\a_m\end{bmatrix}\sim N\left(\begin{bmatrix}0 \\\vdots \\0\end{bmatrix},\begin{bmatrix}\sigma_{a_1}^2 & \cdots & \operatorname{cov} \\\vdots & \ddots & \vdots \\\operatorname{cov} & \cdots & \sigma_{a_m}^2\end{bmatrix}\otimes G\right)$$ and $$\begin{bmatrix}e_1 \\\vdots \\e_m\end{bmatrix}\sim N\left(\begin{bmatrix}0 \\\vdots \\0\end{bmatrix},\begin{bmatrix}W_1 & & 0 \\& \ddots & \\0 & & W_m\end{bmatrix}\begin{bmatrix}\sigma_{e_1}^2 & & \operatorname{cov} \\& \ddots & \\\operatorname{cov} & & \sigma_{e_m}^2\end{bmatrix}\right)$$

## Results

### The impact of data requirements on variance components and prediction ability

**Fig. 2:**
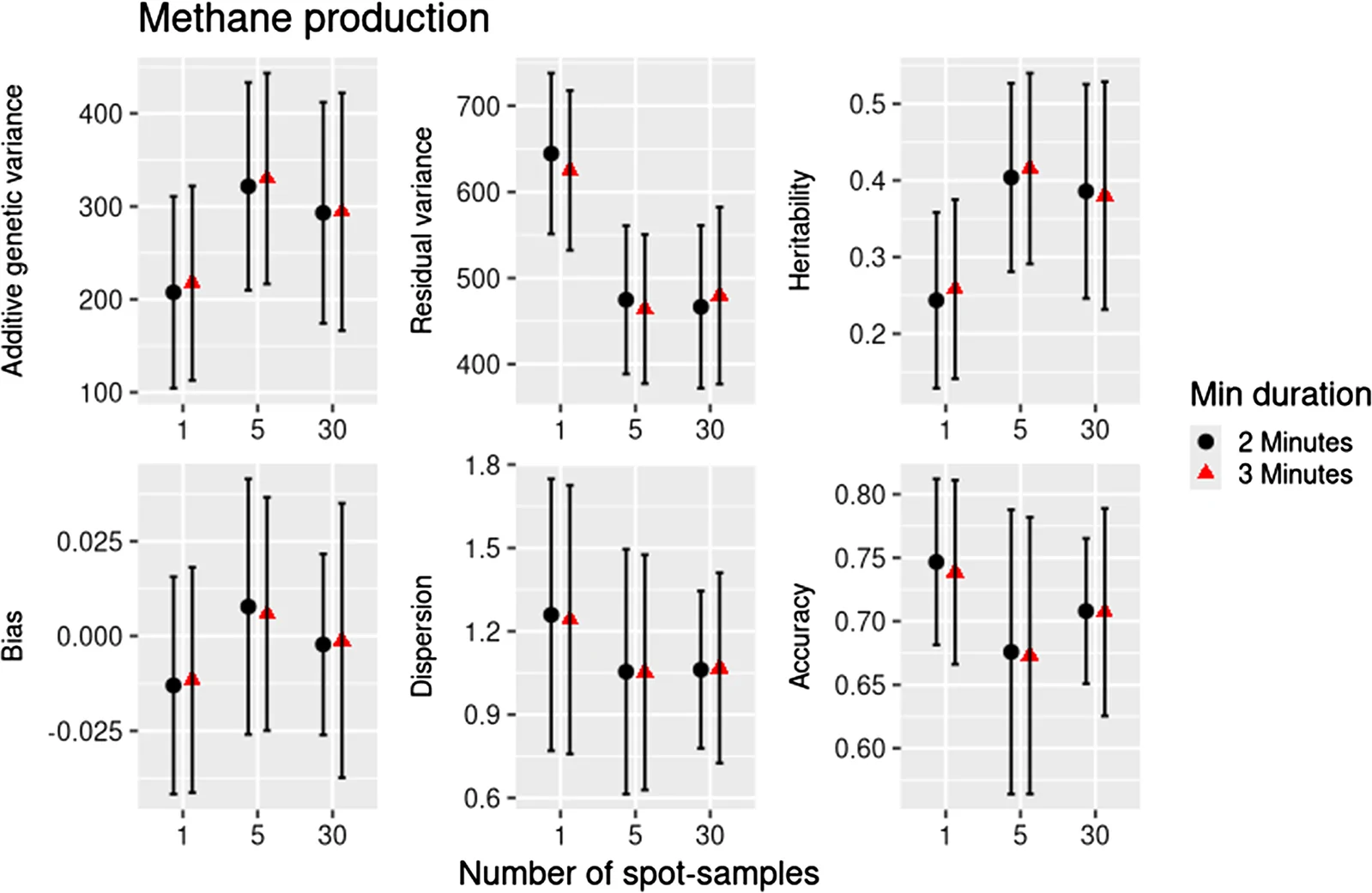
Trends in additive genetic variance (top left), residual variance (top middle), and heritability (top right) of methane production, as well as bias (bottom left), dispersion (bottom middle) and accuracy (bottom right) of genomic estimated breeding values for methane production, in the scenarios including animals with one, five or 30 spot-samples of at least two- (black circles) or three-minute (red triangles) duration

There was a trend that including phenotypes from animals with at least one spot-sample resulted in lower additive genetic variances and higher residual variances, and consequently lower heritability estimates, compared to more stringent requirements for number of spot-samples (Fig. 2, Additional File 3 Tables S2-4). However, there were no substantial difference in variance components between the scenarios requiring animals to have a minimum of five, ten, 15, 20, 25 or 30 spot-samples in order to have their phenotype included in the analysis. Editing data based on minimum duration of spot-samples of two- or three-minute did not have systematic impacts on the variance components when examined across traits.

The cross-validation showed no significant differences in bias, dispersion or accuracy of GEBVs between scenarios within trait (Fig. 2, Additional File 3 Tables S5-7). There were also no significant bias or over-/under-dispersion of GEBVs for any scenario or trait. The SEs of the dispersion tended to decrease as the data requirements became more stringent.

As the variance components and prediction stabilised once individuals had at least five spot-records, this threshold was used for estimation of variance components and correlations with other traits in the following sections.

### Variance components

All traits had additive genetic variances significantly different from zero based on their 95% confidence intervals (Table 3). The emission traits had moderate heritabilities ranging from 0.24 (CH_4−Yld_) to 0.43 (CO_2−Prd_). The production traits were moderately to highly heritable.

The variances of the residual emission traits were lower than those of CH_4−Prd_, particularly the additive genetic variances. The additive genetic variances of CH_4−ResDFI_, CH_4−ResADG_ and CH_4−ResMWT_ was 66%, 79% and 67%, respectively, of the additive genetic variance of CH_4−Prd_. The residual variances of CH_4−ResDFI_, CH_4−ResADG_ and CH_4−ResMWT_ was 89%, 87% and 88%, respectively, of the residual variance of CH_4−Prd_.

**Table 3:** Estimated variance components from the scenario requiring animals to have five or more two-minute or longer duration spot-samples, as well as the production traits

Trait	$\:{\boldsymbol{\sigma\:}}_{\boldsymbol{a}}^{2}$	$\:{\boldsymbol{\sigma\:}}_{\boldsymbol{e}}^{2}$	$\:{\boldsymbol{h}}^{2}$
**Emission traits**			
CH_4-Prd_	321.58 (56.96)	474.74 (43.91)	0.40 (0.06)
CH_4-ResDFI_	208.28 (48.73)	536.58 (56.96)	0.28 (0.06)
CH_4-ResADG_	268.34 (55.99)	560.40 (110.64)	0.32 (0.08)
CH_4-ResMWT_	211.96 (46.93)	503.78 (45.43)	0.30 (0.06)
CH_4-Int_	0.0006 (0.0001)	0.0015 (0.0001)	0.29 (0.06)
CH_4-Yld_	0.76 (0.21)	2.46 (0.19)	0.24 (0.06)
CO_2-Prd_	0.38 (0.07)	0.51 (0.05)	0.43 (0.06)
**Production traits**			
DFI	1.08 (0.15)	1.29 (0.11)	0.45 (0.05)
ADG	0.02 (0.00)	0.06 (0.00)	0.24 (0.05)
MWT	1198.10 (138.50)	862.70 (90.98)	0.58 (0.05)

$\:{\sigma\:}_{a}^{2}$=additive genetic variance,$\:{\sigma\:}_{e}^{2}$= residual variance,$\:{h}^{2}$= heritability, CH_4-Prd_ = methane production (g/day), CH_4-ResDFI_ = residual methane production corrected for daily feed intake (g/day), CH_4-ResADG_ = residual methane production corrected for average daily gain (g/day), CH_4-ResMWT_ = residual methane production corrected for mid-test weight (g/day), CH_4-Int_ = methane intensity (g/day / kg), CH_4-Yld_ = methane yield (g/day / kg/day), CO_2-Prd_ = carbon dioxide production (kg/day), DFI = daily feed intake (kg/day), ADG = average daily gain (kg/day), MWT = mid-test weight (kg)

### Correlations between traits

The CH_4_ traits were moderately positive to very highly positively correlated to each other (Table 4), with a range between 0.79 (CH_4−Prd_ and CH_4−Yld_) and 0.93 (CH_4−ResDFI_ and CH_4−ResADG_). The genetic correlations between CO_2−Prd_ and CH_4−Prd_ or CH_4−ResADG_ were moderately positive (0.64 and 0.46, respectively), while the genetic correlations between CO_2−Prd_ and the remaining CH_4_ traits were generally low (0.06–0.23).

**Table 4:** Estimated genetic correlations between the emission traits

	CH_4-Prd_	CH_4-ResDFI_	CH_4-Res ADG_	CH_4-ResMWT_	CH_4-Int_	CH_4-Yld_
CH_4-ResDFI_	0.82 (0.06)					
CH_4-ResADG_	0.92 (0.06)	0.93 (0.04)				
CH_4-ResMWT_	0.80 (0.06)	0.87 (0.07)	0.81 (0.09)			
CH_4-Int_	0.85 (0.04)	0.89 (0.05)	0.85 (0.07)	*		
CH_4-Yld_	0.76 (0.07)	*	0.91 (0.06)	0.85 (0.06)	0.85 (0.06)	
CO_2-Prd_	0.64 (0.08)	0.12 (0.12)	0.46 (0.12)	0.19 (0.11)	0.23 (0.14)	0.06 (0.17)

CH_4-Prd_ = methane production (g/day), CH_4-ResDFI_ = residual methane production corrected for daily feed intake (g/day), CH_4-ResADG_ = residual methane production corrected for average daily gain (g/day), CH_4-ResMWT_ = residual methane production corrected for mid-test weight (g/day), CH_4-Int_ = methane intensity (g/day / kg), CH_4-Yld_ = methane yield (g/day / kg/day), CO_2-Prd_ = carbon dioxide production (kg/day)

*Model failed for the model including CH_4-Yld_, CH_4-Prd_ and daily feed intake and the model including CH_4-Int_, CH_4-Prd_ and mid-test weight as the G matrix was not positive definite

The genetic correlation was moderately positive between ADG and MWT (0.48) and highly positive between DFI and either ADG (0.82) or MWT (0.82) (Table 5). The genetic correlations between the production traits and CO_2−Prd_ were moderately to highly positive (0.54–0.88), and higher than those between CH_4−Prd_ and the production traits (0.39–0.60). Compared to CH_4−Prd_, the CH_4_ efficiency traits had lower genetic correlations with the production traits (-0.27 to 0.26).

**Table 5:** Estimated genetic correlations between the production traits and between the production and emission traits

	DFI	ADG	MWT
ADG	0.82 (0.08)		
MWT	0.75 (0.05)	0.48 (0.10)	
CH_4−Prd_	0.58 (0.09)	0.39 (0.14)	0.60 (0.08)
CH_4−ResDFI_	0.00 (0.00)	-0.07 (0.14)	0.18 (0.09)
CH_4−ResADG_	0.26 (0.12)	0.00 (0.00)	0.14 (0.16)
CH_4−ResMWT_	0.18 (0.10)	0.14 (0.16)	0.00 (0.00)
CH_4−Int_	0.18 (0.13)	0.12 (0.18)	0.05 (0.12)
CH_4−Yld_	-0.10 (0.14)	-0.27 (0.19)	0.13 (0.13)
CO_2−Prd_	0.88 (0.05)	0.54 (0.12)	0.82 (0.05)

DFI= daily feed intake (kg/day), ADG= average daily gain (kg/day), MWT = mid-test weight (kg), CH_4-Prd_ = methane production (g/day), CH_4-ResDFI_ = residual methane production corrected for daily feed intake (g/day), CH_4-ResADG_ = residual methane production corrected for average daily gain (g/day), CH_4-ResMWT_ = residual methane production corrected for mid-test weight (g/day), CH_4-Int_ = methane intensity (g/day / kg), CH_4-Yld_ = methane yield (g/day / kg/day), CO_2-Prd_ = carbon dioxide production (kg/day)

## Discussion

### Data requirements for greenfeed emission phenotypes

The current study has illustrated that genetic evaluation models used to estimate breeding values and variance components for emission traits recorded using GreenFeeds enable the use of phenotypes based on five or more spot-samples of two-minute or longer duration without compromising the quality of the outputs of such models. Previous studies that have examined the repeatability of methane emissions have suggested that a minimum of 30 three-minute or longer duration spot-samples per animal was required to maximise repeatability of phenotypes [11]. Compared to requiring at least 30 three-minute spot-samples, using a minimum of five two-minute or longer duration spot-samples meant that 35% more animals were evaluated, hence increasing the size of the reference population for genomic prediction.

The lack of differences observed between a minimum of five spot-samples and more stringent thresholds may have been impacted by the weighting strategy used. To account for the differing volumes of information used to define the mean phenotypes the residuals were weighted based on the number of spot-samples. The function used to calculate the weighting for the residuals followed an asymptotic curve (Additional File 2, Figure S1). This function therefore accounted for the diminishing increase in precision of the trial average with increasing number of spot-samples, i.e. there is a larger increase in precision of an average based on 10 values relative to one based on 5 values, vs. a mean based on 55 values relative to one based on 50 values. This enabled the model to emphasise the more accurate phenotypes of animals with more spot-samples, without overemphasising animals with a very high number of spot-samples, and to adjust the more uncertain phenotypes of animals with few spot-samples through their genomic relationships.

The current study should not be viewed as a guide for when to stop sample collection, rather how to optimise use of data that has been recorded. More research is warranted into the appropriate length of emissions recording trials when taking the variable number of spot-samples into consideration. The data used in this study was collected over trials of variable lengths and some trial groups were prolonged when machine availability allowed for it. In a commercial setting this will likely not be possible and knowing how long equipment is necessary per trial group may help optimize the efficiency of data collection.

### Heritabilities and genetic correlations

All emission traits had significant additive genetic variance and heritabilities, illustrating a potential for selecting genetically lower emitting animals to reduce greenhouse gas emissions from Australian beef cattle. The variance components of, and relationships among, all traits will be discussed here, focusing on the results from the dataset where animals were required to have five two-minute or longer duration spot-samples.

Variance components of emission traits in Australian beef cattle obtained from data collected with GreenFeed units have not been published previously. However, heritabilities of 0.39–0.43 (SE 0.09–0.11) for CH_4−Prd_ and 0.50 (SE 0.09–0.11) for CO_2−Prd_ have been reported in Irish beef cattle recorded using GreenFeed units [8, 9]. These heritabilities were higher than those found in the current study, particularly for CO_2−Prd_. Differences between Irish and Australian production systems, breeds of animals in the populations and the type of cattle recorded (steers vs. bulls, heifers and steers) may impact comparisons between this study and that of Ryan et al. [8]. Another contributing factor could be that adjustments for the diurnal pattern, machine effects and number of spot-samples that contributed to the trial average were used in this study compared to an unadjusted trial average as used by Ryan et al. [8].

Heritabilities of emission traits in Australian beef cattle have previously been reported from a dataset of 1096 Angus animals recorded using respiration chambers as part of the Australian National Methane Livestock Program (NLMP, 2012–2015) [4–7]. The current study found similar heritabilities of CH_4−Int_ and CH_4−Yld_, but higher heritability of CH_4−Prd_ and the CH_4−Res_ traits and lower heritability of CO_2−Prd_, compared to those reported on the NLMP dataset. The differences in heritabilities may be due to differences in population structure (Angus vs. multibreed) and recording methods. Respiration chambers, as used in the NLMP, are an accurate method to measure emissions over short periods as they capture all emissions across the full recording period (usually 24- or 48-hours) for all animals [32]. In contrast, GreenFeed units, as used in the current study, record spot-samples of attending animals over longer periods. Consequently, variance components of emission production captured by respiration chambers and GreenFeed units are likely to show some differences.

The emission production traits (CH_4−prd_ and CO_2−prd_) had stronger relationships to DFI and MWT than ADG. Ryan et al. [9] likewise reported that emission production traits were more strongly genetically correlated to feed intake and carcass weight than age at finishing. Similarly, CH_4−ResADG_ had a smaller relative reduction in additive genetic variances and a higher heritability than CH_4−ResDFI_ and CH_4−ResMWT_. This indicates that growth of the animals has less impact on emission than the feed intake and weight of the animals.

The estimated heritability of CO_2−Prd_ was slightly higher than that of CH_4−Prd_ in the current study. Similarly, previous studies have found higher heritabilities of CO_2−Prd_ than CH_4−Prd_ [7–9]. The tendency for higher heritabilities of CO_2−Prd_ could be due to CO_2−Prd_ being genetically more correlated to feed intake, growth and weight traits than CH_4−Prd_, which was observed in both the current study and in the studies by Donoghue et al. [7] and Ryan et al. [9]. This pattern indicates that the feed intake, growth and size of the animals is more highly related to CO_2_ emissions than CH_4_ emissions. Since the production traits were found to be moderately to highly heritable traits themselves, the fact that their variation relates more to the variation of CO_2−Prd_ than CH_4−Prd_ could be part of why CO_2−Prd_ generally have higher heritability than CH_4−Prd_.

The highly positive genetic correlation between CO_2−Prd_ and DFI also indicates that CO_2−Prd_ could be used as a proxy for DFI in cases where DFI records are not obtainable, such as for animals on pasture. In beef cattle, genetic correlations of 0.76–0.88 (SE 0.03–0.08) and 0.95 (SE 0.03) between CO_2_ production and dry matter intake have been reported for GreenFeed [8, 9] and respiration chamber [7] recorded CO_2_ production, respectively. Furthermore, Huhtanen and Bayat [33] found that residual CO_2_ production explained 57% of the phenotypic variation in residual metabolizable energy intake in dairy cows fed a grass silage-based diet. However, these studies were not conducted during pasture grazing, and further research is necessary to understand the relationship between CO_2_ production and feed intake on pasture.

### Implications of trait definitions for emission traits

The nature of ratio traits can make direct selection on CH_4−Int_ or CH_4−Yld_ challenging. Ratios based on correlated component traits have a closed approximate form [34] and a non-normal distribution [35] and it has been shown that direct selection on ratio traits based on correlated component traits can place disproportionate selection pressure on the component traits, resulting in unpredictable selection responses in the traits [35, 36]. As observed in the current study, as well as previous studies, CH_4_ production, feed intake and live weight traits have moderately to highly positive genetic correlations in Australian beef cattle [4, 6, 7]. Thus, direct selection on the ratio traits may result in selection responses in the component traits that deviate from what is expected based on their genetic correlations. Direct selection on CH_4−Int_ or CH_4−Yld_ may therefore not be the optimal strategy to improve CH_4_ efficiency.

Having ratio traits based on correlated component traits can also make evaluations challenging, particularly for multi-trait analysis fitting both the ratio and the component traits. In the current study, both CH_4−Int_ and CH_4−Yld_ were fitted in trivariate models with their component traits to obtain genetic correlations between the ratio traits and the CH_4−Res_ traits. Both the model fitting CH_4−Int_ with its component traits and the model fitting CH_4−Yld_ and its component traits had non-positive definite covariance matrices. This was likely due to the fact that one of the traits was directly derived from the other two traits.

The issues regarding selection on ratio traits have often been discussed when trying to define feed efficiency using feed conversion ratio (feed intake/body weight gain). One of the proposed solutions has been to use residual (or net) feed intake as a measure of feed efficiency instead [21]. Residual feed intake is typically a feed intake trait (DFI, dry matter intake etc.) adjusted for one or more productivity traits [21]. The variances obtained post-adjustment represent the variation in feed intake that is not caused by the variation in productivity. Selecting to reduce residual feed intake would therefore reduce the ‘wasted’ portion of the ingested feed, resulting in increased efficiency.

Residual emission traits have been suggested for selection to improve emission efficiency in livestock as an alternative to the ratio traits, using the same principles as residual feed intake. Based on the variance and heritabilities found in the current study, single trait selection on the residual CH_4_ traits would yield less direct selection response than selection on CH_4−Prd_ but would have no correlated response in the trait used in the adjustment for the residual CH_4_ trait. Furthermore, residual traits can be adjusted for more than one correlated trait simultaneously to ensure none of the adjustment-traits have a correlated response to single trait selection on the residual trait [21]. This was not done in the current study, as the goal here was to understand the relative impact of removing the variation caused by each of the recorded production traits on the variation of CH_4−Prd_. However, including residual traits, whether they be residual emission or residual feed intake traits, in selection indexes is equivalent to including the emission trait itself provided the weightings of the other traits in the index are updated accordingly [21]. Thus, if done correctly, inclusion of emission production traits or residual emission traits in selection indexes will result in the same selection outcomes.

The emission production traits have the benefit of being the most readily available traits out of the emission traits defined in the current study, as these are obtained directly from C-Lock Inc when using GreenFeed units to record emissions and do not necessitate recording of (trial) weights or individual feed intake. While weights are generally routinely recorded in most beef breeding programs, individual feed intake can be challenging to record, particularly in extensive, pasture based, production systems. Of the traits defined in the current study the emission production traits may therefore be the most suitable traits for genetic evaluation when implemented in a balanced selection index such that progress can be achieved in both productivity and emissions.

## Conclusion

The results presented here showed that requiring animals to have at minimum five spot-samples of at least two minutes duration were appropriate data requirements for the emission data currently available on Australian feedlot beef cattle. This data requirement resulted in models that performed better than models using data where all animals with at least one spot-sample were included, regardless of minimum required duration. However, more stringent requirements for number and/or duration of spot-samples did not result in models that performed systematically differently to models using data requiring a minimum of five two-minute duration spot-samples. This was true for all examined emission traits.

The results further showed that selection to reduce emissions in beef cattle should be possible when selecting on emission traits from feedlot animals recorded with GreenFeed units. The emission traits had low to moderate heritabilities. Adjusting CH_4_ for feed intake or weight (either as a ratio or a residual trait) resulted in reasonable additive genetic variances and it could be possible to reduce CH_4_ emissions without changing the feed intake or weight of the animals. In contrast, feed intake and liveweight were found to have stronger genetic relationships with CO_2−Prd_ than CH_4−Prd_, and selecting to reduce CO_2−Prd_ without impacting the animals’ productivity could be challenging.

## Supplementary Information

Below is the link to the electronic supplementary material.

Supplementary Material 1

Supplementary Material 2

Supplementary Material 3

## Data Availability

Any data request should be made directly to Meat and Livestock Australia.
