# Supplementary material for: The genetic relationships between different definitions of emissions outputs and feedlot production traits
Source: Genet Sel Evol. 2026 Sep 11;58:65. doi: 10.1186/s12711-026-01086-1 (PMC13570512; doi:10.1186/s12711-026-01086-1)
Supplement: Supplementary file 1 [file 12711_2026_1086_MOESM2_ESM.docx]

**Additional file 2 – Calculations of residual weights**

The emission records were analysed with weighted residuals to account for the difference in information available per animal, i.e. the number of spot-samples ($n_{s})$ used to calculate the phenotypic average. The weighting of the residuals results in more emphasis being placed on phenotypes based on more spot-samples. When using weighted residuals, it is important to ensure that the function used to calculate the weights ($w$) is an appropriate reflection of the relative increase in certainty of the trial average phenotype(s) being analysed. In this study, three different functions based on $n_{s}$ were tested: 1) $w=n_{s}$, 2) $w=\sqrt{n_{s}}$, and 3) $w=1-\frac{1}{n_{s}}$.

The simplest weight function was $w=n_{s}$, which results in a linear increase in emphasis with increasing $n_{s}$. For this function, the difference between the emphasis placed on a phenotype based on $n_{s}$*vs* $n_{s}+1$ is constant no matter the size of $n_{s}$ (Figure S1). Meanwhile both $w=\sqrt{n_{s}}$ and $w=1-\frac{1}{n_{s}}$result in non-linear increases in emphasis as $n_{s}$ increased. Thus, the difference between the emphasis placed on a phenotype based on $n_{s}+1$ *vs* a phenotype based on $n_{s}$ depends on the size of $n_{s}$ for both $w=\sqrt{n_{s}}$ and $w=1-\frac{1}{n_{s}+1}$. When $n_{s}$ is low the addition of a single spot-sample will have a larger impact on the weight compared to when $n_{s}$ is higher. The idea of using $w=\sqrt{n_{s}}$ was derived for the standard error of means function ($SEM=\frac{SD}{\sqrt{n}}$). The $w=1-\frac{1}{n_{s}+1}$ function stemmed from the effective number of records in a contemporary group, but treating each animal as the contemporary group. To avoid weights of zero (i.e. no emphasis on the phenotype and residuals being regressed towards zero) for animals with only one spot-sample, 1 was added to the denominator.

The three weight functions were tested for the univariate models for all 14 scenarios for methane and carbon dioxide production, intensity and yield as well as daily feed intake. The likelihood of the three different weighted models, as well as an unweighted model, was compared within trait and scenario to determine the best fitting model. This is possible as the datasets, fixed effects and the number of estimated parameters remain the same within trait and scenario regardless of weight factor. Based on the likelihood $w=1-\frac{1}{n_{s}+1}$ was the best fit of the tested weights. This was likely due to $1-\frac{1}{n_{s}+1}$ better reflecting the relative impact of adding a single spot-sample when n_s_ is low *vs* when n_s_ is high.

**Figure S1. The weight factor based on** $\boldsymbol{n}_{\boldsymbol{s}}$ **(solid black line),** $\sqrt{\boldsymbol{n}_{\boldsymbol{s}}}$ **(dotted red line) and** $\boldsymbol{1-}\frac{\boldsymbol{1}}{\boldsymbol{n}_{\boldsymbol{s}}\boldsymbol{+1}}$ **(dashed green line). The weights were scaled to be between 0 and 1 for the plot.**
